# Evaluation of KRAS and NRAS mutations in metastatic colorectal cancer: an 8‐year study of 10 754 patients in Turkey

**DOI:** 10.1002/1878-0261.70042

**Published:** 2025-05-23

**Authors:** Gozde Kavgaci, Izzet Akiva, Yavuz Hakan Ozon, Hakan Berkil, Huseyin Karadayi, Omer Dizdar, Suayib Yalcin

**Affiliations:** ^1^ Department of Medical Oncology Hacettepe University Cancer Institute Ankara Turkey; ^2^ Heliks R&D and Biotechnology Inc. Istanbul Turkey; ^3^ Genetiks Genetic Diagnostic Center Istanbul Turkey

**Keywords:** KRAS, metastatic colorectal cancer, minisequencing, NRAS, real‐world data

## Abstract

This study aimed to evaluate KRAS and NRAS mutations over an eight‐year period to provide a comprehensive understanding of the genetic landscape of metastatic colorectal cancer (mCRC) in Turkish patients. Tumor tissue samples were collected from 10 754 patients with mCRC between January 2015 and June 2023 from multiple centers across Turkey, and all genetic analyses were performed at a single facility. DNA was analyzed for mutations in codons 12, 13, 59, 61, 117, and 146 of the KRAS and NRAS genes using nested PCR and multiplex minisequencing techniques. Among the conclusive results from 10 681 patients, 46.6% (4982) had KRAS mutations, and 53.4% (5699) were wild‐type. The most common KRAS mutations were G12D (30.3%), G12V (20.1%), G13D (18.4%), and G12C (7.0%). Results from the 5699 KRAS wild‐type patients revealed that 480 (8.5%) had NRAS mutations, while 91.5% were NRAS wild‐type. The most frequent NRAS mutations were Q61K (19.7%), G12D (19.1%), and G12V (12%). This study provides a large‐scale, real‐world dataset of KRAS and NRAS mutation profiles in Turkish mCRC patients, contributing significantly to the understanding of the genetic characteristics of mCRC in this population.

## Graphical abstract

This nationwide study evaluated KRAS and NRAS mutations in 10 754 Turkish patients with metastatic colorectal cancer. The results revealed a mutation frequency of 51.1%, with 46.6% having KRAS mutations, 4.5% having NRAS mutations, and 48.5% being wild‐type for both. These findings provide a comprehensive overview of the molecular landscape of metastatic colorectal cancer in Turkey.

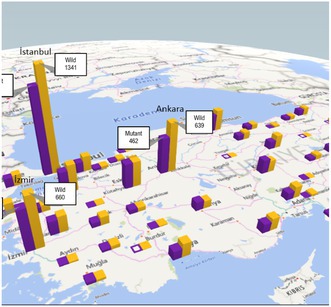

**Abbreviations**
COSMIC: catalogue of somatic mutations in cancer
CRC: colorectal cancer
ddNTP: dideoxynucleotide
DNA: deoxyribonucleic acid
dNTP: deoxynucleoside triphosphate
FM: Foundation Medicine
MAPK: mitogen‐activated protein kinase
mCRC: metastatic colorectal cancer
OS: overall survival
PCR: polymerase chain reaction
PFS: progression‐free survival
PI3K: phosphoinositide 3‐kinase

## 1 Introduction

Colorectal cancer (CRC) is the third most commonly diagnosed cancer worldwide, with more than 1.9 million new cases annually [1]. It is also the second leading cause of cancer‐related death, accounting for approximately 900 000 deaths each year [1]. Approximately 20–40% of patients with CRC develop synchronous or metachronous metastases, and the 5‐year survival rate for metastatic CRC (mCRC) is only 15.7% [2, 3].

Due to the heterogeneity of mCRC, the role of targeted therapy in its treatment has increased significantly, and molecular analysis has become essential to guide treatment decisions [4, 5]. RAS GTPases, including KRAS and NRAS, are among the most frequently mutated oncogenes in cancer, with KRAS being the most commonly mutated isoform [6]. These mutations are often single nucleotide point mutations, most commonly occurring in exon 2 (codons 12 and 13) and exon 3 (codon 61) [7]. Mutations cause the proteins to remain in a persistently activated state, resulting in continuous cell signaling and reduced GTPase activity. The signaling pathways activated by RAS include the Raf/MEK/ERK mitogen‐activated protein kinase (MAPK) pathway and the phosphoinositide 3‐kinase (PI3K)/Akt pathway. Deregulated RAS signaling increases cell proliferation, angiogenesis, migration, and metastasis while decreasing apoptosis [6, 8].

KRAS mutations are found in approximately 45% of mCRC tumors, while NRAS mutations occur in approximately 3–5% of these tumors [9]. KRAS exons 2, 3, and 4 and NRAS exons 2, 3, and 4 mutations serve as negative predictive biomarkers for poorer overall survival (OS) and are associated with resistance to antiepidermal growth factor receptor (EGFR) antibodies such as cetuximab and panitumumab because the mutations result in continued activation of the MAPK pathway even when EGFR is inactivated by drugs [10, 11, 12, 13, 14, 15]. This study, conducted over 8 years in Turkey, is evaluating KRAS and NRAS mutations in 10 754 mCRC samples. The aim was to improve the understanding of the genetic landscape of mCRC in Turkish patients, reflecting real‐world data, and thereby guide more effective treatment strategies.

## 2 Materials and methods

### 2.1 Study population and mutation analysis

This study included tumor tissue samples from adult patients (≥ 18 years old at diagnosis) diagnosed with metastatic colorectal cancer (mCRC) between January 1, 2015, and June 30, 2023. A total of 10 754 anonymized samples were collected from multiple centers across Turkey. All genetic analyses were performed at a single facility—Genetiks Genetic Diagnostic Center (Istanbul, Türkiye)—using a standardized methodology.

The mutation analysis focused on mutations in the KRAS and NRAS genes. Mutations were evaluated at codons 12, 13, 59, 61, 117, and 146. Two rounds of nested polymerase chain reaction (PCR) followed by multiplex minisequencing were performed for the second, third, and fourth exons of the KRAS/NRAS genes.

The study was conducted in accordance with the Declaration of Helsinki and approved by the Hacettepe University Health Sciences Research Ethics Board and Commissions (SBA24/592). The requirement for written informed consent was waived by the ethics committee, as the study involved the analysis of anonymized data without any direct patient intervention. The Genetiks Genetic Diagnostic Center database used in this study is fully anonymized and complies with all relevant regulations for protecting patient privacy.

### 2.2 DNA extraction

Nested PCR was used as the deoxyribonucleic acid (DNA) amplification technique. This method involves two consecutive rounds of PCR using two sets of primers. The first round targets the outer region of the DNA of interest, and the resulting product is then used as a template for the second round of PCR with inner primers specific for the inner region of the target DNA. This nested PCR approach allows for more total cycles while minimizing the amplification of unspecific products. The likelihood of unwanted PCR products containing binding sites for both sets of primers is minimal, reducing contamination from primer dimers, hairpins, and alternative primer target sequences.

In the first round of PCR, the total reaction volume was 50 μL, including 10 pmol of each outer primer. The reaction conditions consisted of 30 PCR cycles, with a denaturation step of 30 s at 94 °C, annealing for 30 s at 55 °C, and extension for 30 s at 72 °C. Each round of PCR began with an initial denaturation step of 5 min at 94 °C and ended with a final extension step of 10 min at 72 °C. For the second round of DNA amplification, 1.5 μL of primary PCR product was added to the reaction mix along with 10 pmol of each inner primer. Reaction conditions for the second round were identical to the first round. Successful amplification was monitored by electrophoresing 5 μL of each PCR product for 15 min at 250 V on a 2% agarose gel in 1× Tris‐borate/EDTA buffer, stained with 0.5 μg·mL^−1^ ethidium bromide.

### 2.3 Minisequencing analysis

After nested PCR, a purification step was performed to remove unbound primers and residual deoxynucleoside triphosphates (dNTPs). The minisequencing reaction was then performed using the ABI Prism SnaPshot Multiplex Kit (Applied Biosystems, Waltham, MA, USA), starting with 10 ng of purified PCR product. The total reaction volume was 4 μL, including 10 pmol of each minisequencing primer. The reaction conditions consisted of 25 PCR cycles, with a denaturation step of 10 s at 96 °C, annealing for 10 s at 50 °C, and extension for 30 s at 60 °C.

In the minisequencing technique, a primer extension reaction was performed using fluorescently labeled dideoxynucleotides (ddNTPs) (ddATP, ddGTP, ddCTP, and ddTTP) complementary to the variant base in the template. A specific primer designed to anneal directly adjacent to the mutation site ensured that incorporation occurred only at that site. Successive rounds of extension and termination produced a fluorescently labeled fragment for analysis. After the primer extension reaction, 1 μL of the minisequencing product was mixed with 10 μL of Hi‐Di formamide (Applied Biosystems). The samples were separated by capillary electrophoresis on an automated DNA sequencer using POP‐7 polymer and 36 cm × 50 μm capillaries. This allowed reliable differentiation of mutation sites among homozygous wild‐types, homozygous mutants, or heterozygotes based on the incorporation of dye‐labeled ddNTPs.

The genemapper analysis software (Thermo Fisher Scientific, Waltham, MA, USA) was used to analyze the base positions and their peak signals, using the dye color to identify the nucleotide of interest. In the minisequencing technique, colors were assigned to individual ddNTPs as follows: green for A, black for C, blue for G, and red for T. Depending on the genotype at the locus, the minisequencing reaction produced one peak (homozygote) or two peaks (heterozygote).

## 3 Results

A total of 10 754 patient samples were collected. We performed KRAS mutation analysis in 10 754 patients and NRAS mutation analysis in 5699 patients diagnosed with mCRC.

### 3.1 KRAS mutations

**Fig. 1:**
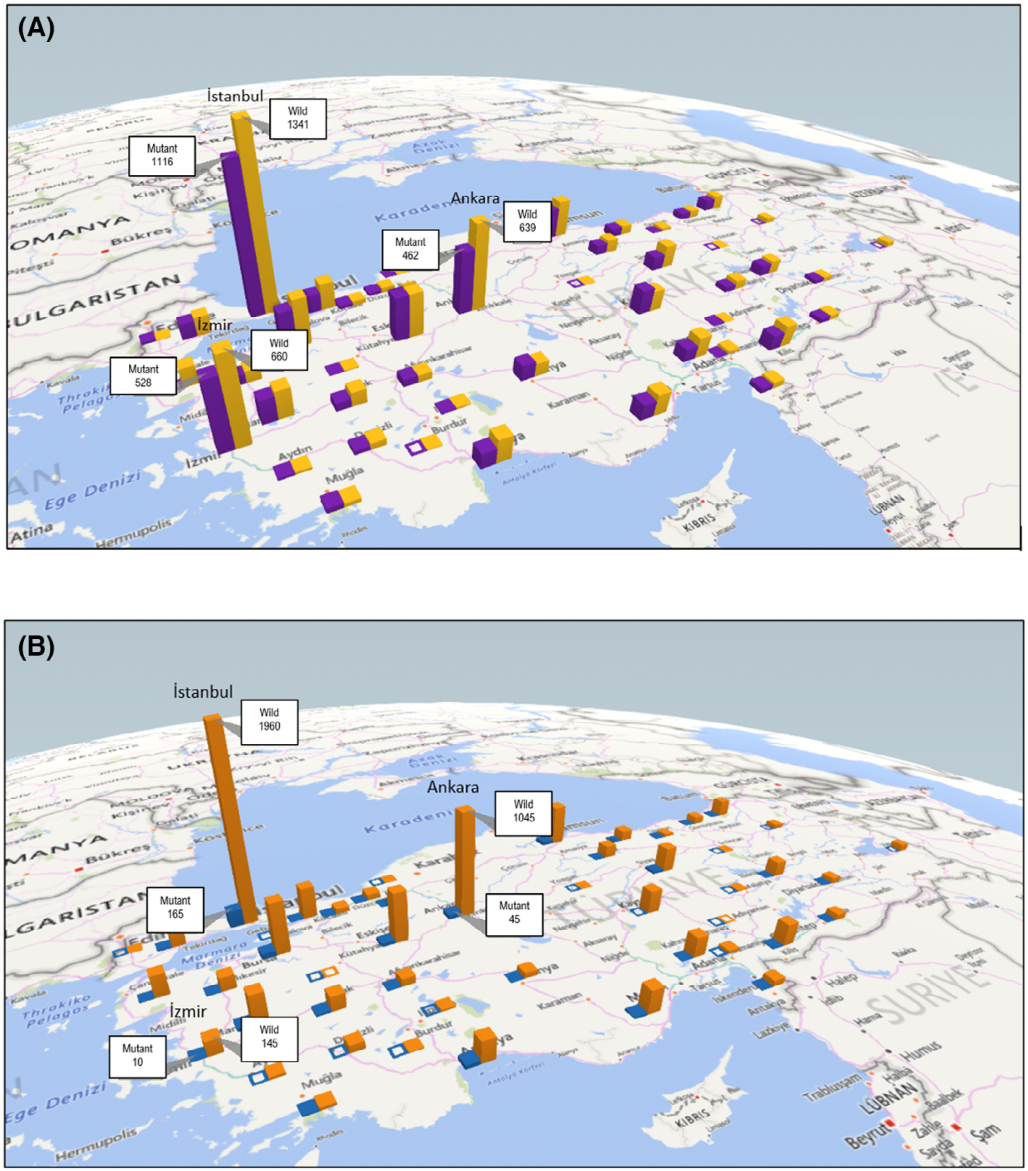
Geographic distribution of KRAS and NRAS mutations across Turkey. (A) Three‐dimensional geographic distribution of KRAS‐mutant (purple) and KRAS wild‐type (yellow) mCRC cases across various cities in Turkey (total *n* = 10 681; mutant *n* = 4982, wild‐type *n* = 5699). The height of each bar indicates the number of patient samples from each city, with major metropolitan areas (Istanbul, Ankara, Izmir). (B) Three‐dimensional geographic distribution of NRAS‐mutant (blue) and NRAS wild‐type (orange) mCRC among KRAS wild‐type patients across various cities in Turkey (total *n* = 5638; mutant *n* = 480, wild‐type *n* = 5158). The height of each bar corresponds to the number of patient samples analyzed from each city, with major metropolitan areas (Istanbul, Ankara, Izmir).

KRAS mutation analysis was performed on 10 754 patients. Of these, 73 results were inconclusive. In the conclusive group of 10 681 patients, 46.6% (4982 patients) had KRAS mutations and 53.4% (5699 patients) were wild‐type for KRAS. The most common mutation was KRAS G12D, present in 30.3% (1547/4982) of KRAS mutant cases, followed by KRAS G12V (20.1%, 1027/4982), KRAS G13D (18.4%, 940/4982), and KRAS G12C (7.0%, 359/4982). The majority of KRAS mutations were located in exon 2, particularly in codon 12, which accounted for 66.7% of KRAS mutations. The most common mutation in this codon was KRAS G12D, accounting for 30.3% of KRAS mutations, followed by KRAS G12V (20.1%) and KRAS G12C (7.0%). For codon 13, located in exon 2, the most common mutation was G13D, found in 18.4% of patients with KRAS mutations. For codon 146 in exon 4, the most common mutation was A146T, accounting for 5.3% of KRAS mutations. Other mutations were identified in codons 59, 61, 117, and 146 at lower frequencies. Of the 4982 patients with KRAS mutations, 120 (2.4%) had two different KRAS mutations. In cases of double KRAS mutations, the most common mutation was KRAS G12D, found in 62 of the 120 cases, followed by KRAS G13D, found in 52 of the 120 cases, and G12V, found in 50 of the 120 cases. The most common double mutation combination was KRAS G12D and G13D, observed in 27 (22.5%) of the 120 patients. The distribution of KRAS mutations across codons is presented in Table 1, and the distribution of KRAS mutations in Turkey is illustrated in Fig. 1A.

**Table 1:** Distribution of mutations in KRAS codons 12, 13, 59, 61, 117 and 146.

Exon	Codon	Nucleotide change (amino acid change)	Number of mutations (%)
Exon 2	Codon 12	c.34G>T (G12C)	359 (7.04%)
		c.34G>C (G12R)	52 (1.02%)
		c.34G>A (G12S)	257 (5.04%)
		c.35G>C (G12A)	162 (3.18%)
		c.35G>A (G12D)	1547 (30.3%)
		c.35G>T (G12V)	1027 (20.13%)
	Codon 13	c.37G>T (G13C)	51 (1.00%)
		c.37G>C (G13R)	9 (0.18%)
		c.37G>A (G13S)	41 (0.80%)
		c.38G>C (G13A)	2 (0.04%)
		c.38G>A (G13D)	940 (18.4%)
		c.38G>T (G13V)	12 (0.24%)
Exon 3	Codon 59	c.175G>C (A59P)	0 (0%)
		c.175G>T (A59S)	2 (0.04%)
		c.175G>A (A59T)	38 (0.74%)
		c.176C>G (A59G)	6 (0.12%)
		c.176C>A (A59E)	5 (0.10%)
		c.176C>T (A59V)	13 (0.25%)
	Codon 61	c.181C>A (Q61K)	31 (0.61%)
		c.182A>T (Q61L)	40 (0.78%)
		c.182A>C (Q61P)	3 (0.06%)
		c.182A>G (Q61R)	19 (0.37%)
		c.183A>C (Q61H)	80 (1.57%)
		c.183A>T (Q61H)	37 (0.72%)

Distribution of the KRAS mutations for the six codons analyzed. The percentage of each mutation is given with respect to the total number of KRAS mutations determined. Mutation data were obtained from a total of 4982 patients, 120 of whom had double KRAS mutations.

### 3.2 NRAS mutations

**Fig. 2:**
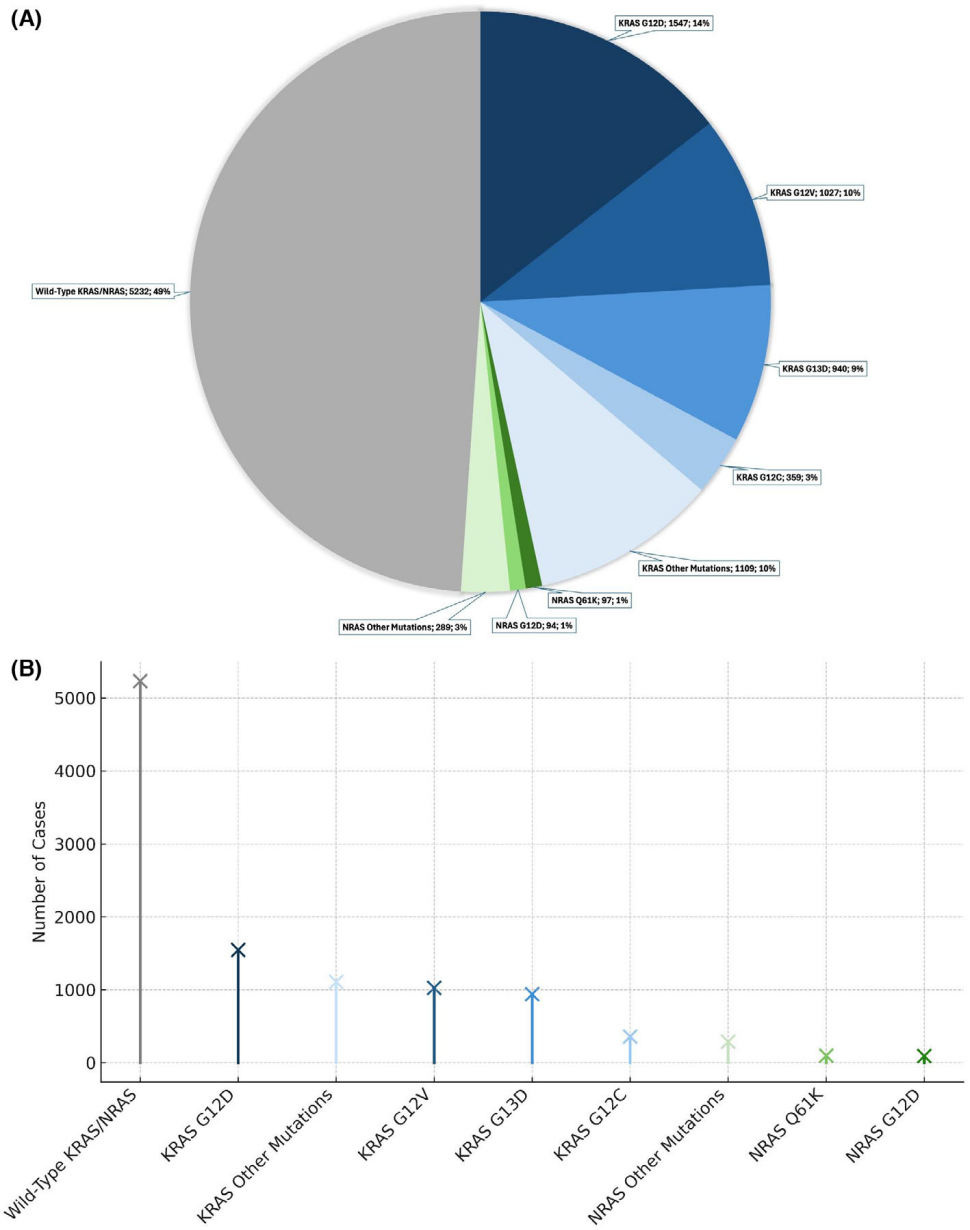
Distribution of KRAS and NRAS mutations among mCRC patients in Turkey. (A) Pie chart illustrating the frequency and proportion of specific KRAS and NRAS mutations and wild‐type cases among 10 681 samples. KRAS mutations (blue shades) include the most common variants: G12D (*n* = 1547, 14%), G12V (*n* = 1027, 10%), G13D (*n* = 940, 9%), and G12C (*n* = 359, 3%), along with other KRAS mutations (*n* = 1109, 10%). NRAS mutations (green shades) include the most frequent variants: Q61K (*n* = 97, 1%), G12D (*n* = 94, 1%), and other NRAS mutations (*n* = 289, 3%). Wild‐type (gray) represents cases that were wild‐type for both KRAS and NRAS mutations (*n* = 5232, 49%). (B) Lollipop plot illustrating the number of specific KRAS and NRAS mutations and wild‐type cases among 10 681 samples.

Mutation analysis of the NRAS gene was performed on the 5699 patients previously identified as wild‐type for KRAS mutations. Among these, 61 NRAS mutation analysis results were inconclusive. Of the remaining patients, 480 (8.4%) were found to have NRAS mutations, while 5158 (90.5%) were wild‐type for NRAS mutations. The most common NRAS mutation was Q61K, present in 19.7% (97/480) of NRAS mutant cases, followed by G12D (19.1%, 94/480), G12V (12%, 59/480), and Q61R (9.5%, 47/480). Among the NRAS mutations observed, codon 61 and codon 12 mutations were the most prevalent, accounting for 42.6% and 39% of NRAS mutations, respectively. In contrast, the contributions from codons 13, 59, 117, and 146 were relatively lower. Thirteen patients exhibited two different NRAS mutations. The most common double mutation combination was NRAS G12 and NRAS A59T, observed in four (30.8%) of the 13 patients. The distribution of NRAS mutations across codons is presented in Table 2, and the distribution of NRAS mutations in Turkey is illustrated in Fig. 1B. The distribution of KRAS and NRAS mutations is illustrated in Fig. 2A,B.

**Table 2:** Distribution of mutations in NRAS codons 12, 13, 59, 61, 117 and 146.

Exon	Codon	Nucleotide change (amino acid change)	Number of mutations (%)
Exon 2	Codon 12	c.34G>T (G12C)	17 (3.45%)
		c.35G>T (G12V)	59 (11.97%)
		c.35G>C (G12A)	10 (2.03%)
		c.35G>A (G12D)	94 (19.06%)
		c.34G>A (G12S)	12 (2.43%)
	Codon 13	c.37G>C (G13R)	12 (2.43%)
		c.37G>A (G13S)	19 (3.85%)
		c.38G>A (G13D)	23 (4.66%)
		c.38G>C (G13A)	1 (0.20%)
		c.38G>T (G13V)	4 (0.81%)
		c.37G>T (G13C)	1 (0.20%)
Exon 3	Codon 59	c.175G>A (A59T)	15 (3.04%)
		c.176C>G (A59G)	1 (0.20%)
		c.176C>A (A59E)	3 (0.61%)
		c.176C>T (A59V)	1 (0.20%)
	Codon 61	c.181C>A (Q61K)	97 (19.67%)
		c.182A>T (Q61L)	41 (8.32%)
		c.182A>G (Q61R)	47 (9.53%)
		c.183A>C (Q61H)	10 (2.03%)
		c.182A>C (Q61P)	4 (0.81%)
		c.183A>T (Q61H)	11 (2.23%)
Exon 4	Codon 117	c.349A>G (K117E)	2 (0.40%)
		c.349A>T (K117)	4 (0.81%)
	Codon 146	c.436G>A (A146T)	5 (1.01%)

Distribution of the NRAS mutations for the six codons analyzed. The percentage of each mutation is given with respect to the total number of NRAS mutations determined. Mutation data were obtained from a total of 480 patients, 13 of whom had double NRAS mutations.

## 4 Discussion

**Fig. 3:**
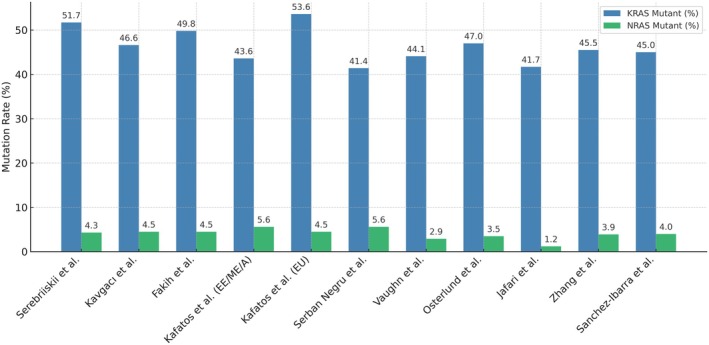
Comparison of KRAS and NRAS mutation frequencies between studies. Bar graph showing KRAS (blue) and NRAS (green) mutation rates (%) from the current study (Kavgacı et al., *n* = 10 681 patient samples) compared with data from previously published international studies [Serebriiskii et al., Fakih et al., Kafatos et al. (Eastern Europe/Middle East/Africa), Kafatos et al. (Europe), Serban Negru et al., Vaughn et al., Osterlund et al., Jafari et al., Zhang et al., and Sanchez‐Ibarra et al.]. Mutation rates are presented as percentages.

In our study, the analysis of 10 754 samples from mCRC patients revealed a mutation frequency of 51.1% in the KRAS and NRAS genes. Among these, 4982 (46.6%) had KRAS mutations, 480 (4.5%) had NRAS mutations, and 5219 (48.5%) were determined to be wild‐type for both KRAS and NRAS (Fig. 3).

In a large dataset of 13 336 CRC patients profiled by Foundation Medicine (FM) Inc. (Boston, MA, USA), KRAS mutations were found in 6896 (51.7%) samples and NRAS mutations in 569 (4.3%) samples, all from metastatic cases [9]. In contrast, the Catalogue Of Somatic Mutations In Cancer (COSMIC) database, which includes more than 75 000 colorectal tumor samples of various stages, reports lower mutation frequencies of 32% for KRAS and 4% for NRAS [16]. The higher mutation frequencies in the FM data are likely due to the exclusive inclusion of metastatic cases and the use of next‐generation sequencing, which detects a wider range of mutations, including those at lower allele frequencies. Therefore, FM data have the highest KRAS mutation rate in the literature with a large real‐world dataset; however, the NRAS rate is similar to that reported in other studies. In contrast, COSMIC collects data from multiple sources using different methodologies such as Sanger sequencing, pyrosequencing, and PCR‐based assays, resulting in variability in mutation detection rates and frequencies.

In the cBioPortal for Cancer Genomics database, of 7590 profiled CRC samples, 3291 (42.1%) had KRAS mutations and 384 (5%) had NRAS mutations [17]. Our mutation frequencies align well with FM and cBioPortal data but are higher than COSMIC due to differences in testing methods, as we analyzed all samples using the same method in a single center and all samples were from patients with mCRC. A pooled analysis of five randomized trials in mCRC by the AIO Colorectal Cancer Study Group included 1239 patients from the FIRE‐1, FIRE‐3, AIOKRK0207, AIOKRK0604, and RO91 trials [11]. Of these, 462 (37.3%) tumors had KRAS mutations and 39 (3.1%) had NRAS mutations, which are lower than those reported in other large datasets. This result may have been influenced by the selection of KRAS exon 2 wild‐type patients in the FIRE‐3 study and the non‐testing of KRAS exons 3–4 and NRAS exons 2–4 in the AIO KRK 0604 and RO91 studies [18, 19]. The higher rates in our data can be attributed to the evaluation of KRAS and NRAS mutations in exons 2, 3, and 4, specifically at codons 12, 13, 59, 61, 117, and 146.

Population‐based studies have shown variable frequencies of KRAS and NRAS mutations among CRC patients across regions. In the United States, Fakih et al. [20] reported that among 6477 mCRC patients, 49.8% had KRAS mutations and 4.5% had NRAS mutations. Vaughn et al. [21] reported similar results in a cohort of 2121 CRC patients, with 44.1% having KRAS mutations and 2.9% having NRAS mutations. However, NRAS codon 12, 13, 61 analysis was only performed in 513 samples that were wild‐type for KRAS codons 12 and 13. In a pooled RAS mutation analysis including Eastern European and Middle Eastern countries, the prevalence of RAS mutations in 4431 tumor samples was 43.6%, without a detailed evaluation of KRAS and NRAS mutations [22]. The prevalence of RAS mutations was lower in Middle Eastern countries, at approximately 33%, and higher in European countries, at 53%. In a separate pooled RAS mutation analysis covering five European countries (France, Germany, Italy, Spain, and the UK), RAS testing was conducted in over 90% of mCRC patients (4109 out of 4455), with a RAS mutation frequency of 53.6% [23].

In the Nordic region, KRAS mutations were observed in 47% and NRAS mutations in 3.5% of 1871 mCRC patients [24]. In North Africa, KRAS mutations were identified in 41.7% and NRAS mutations in 1.2% of 1795 CRC patients. However, both exon 2, 3, and 4 mutations of the KRAS and NRAS genes were analyzed in only 23.5% of the samples, which may explain the lower reported rates of KRAS and NRAS mutations. In Greece and Romania, KRAS mutations were found in 41.4% of 2425 CRC patients [25]. Detailed mutation analysis of KRAS/NRAS (exons 2, 3, and 4) was performed in only 354 patients, revealing NRAS mutations in 5.6%. In China, 45.5% of 1110 CRC patients had KRAS mutations and 3.9% had NRAS mutations [26]. In Mexico, 45% of 500 mCRC patients had KRAS mutations and 4% had NRAS mutations [27]. Detailed KRAS and NRAS mutation analyses according to these studies are presented in Table 3. When comparing KRAS and NRAS mutation frequencies across different population‐based studies, it is apparent that the frequencies are generally similar when KRAS and NRAS exon 2, 3, and 4 mutations are examined in all samples.

**Table 3:** KRAS and NRAS mutation analysis across different studies.

Author	Population	# of patients	KRAS mutant	KRAS G12C	NRAS mutant	Wild‐type for RAS
Serebriiskii et al.	Foundation Medicine	13 336^a^	6896 (51.7%)	N/A	569 (4.3%)	5871 (44%)
Kavgaci et al.	Turkey	10 754^a^	4982 (46.6%)	359 (3.4%)	480 (4.5%)	5219 (48.5%)
Fakih et al.	USA	6477^a^	3230 (49.8%)	238 (3.7%)	289 (4.5%)	2720 (41.9%)
Kafatos et al.	Eastern Europe/Middle East/Argentina	4431	1932 (43.6%)			2499 (56.3%)
Kafatos et al.	France, Germany, Italy, Spain, the UK	4109^a^	2202 (53.6%)			1907 (46.4%)
Serban Negru et al.	Greece and Romania	2425	1005 (41.4%)	N/A	20 (5.6%)	N/A^b^
Vaughn et al.	USA	2121	936 (44.1%)	74 (3.4%)	26 (2.9%)	N/A^c^
Osterlund et al.	Nordic	1871^a^	881 (47%)	103 (5.5%)	66 (3.5%)	821 (43.8%)
Jafari et al.	North Africa	1795	749 (41.7%)	44 (2.4%)	35 (1.2%)	N/A^d^
Zhang et al.	China	1110	504 (45.5%)	24 (2.2%)	43 (3.9%)	563 (50.7%)
Sanchez‐Ibarra et al.	Mexico	500^a^	225 (45%)	17 (3.4%)	20 (4%)	238 (47.6%)

a Metastatic only.

b KRAS/NRAS (exons 2, 3 and 4) analysis was done in only 354 patients.

c NRAS codon 12, 13, 61 analysis was done in only 513 samples wild‐type for KRAS Codons 12 and 13.

d KRAS/NRAS (exons 2, 3 and 4) analysis was done in only 23.5% of the samples.

The KRAS G12C mutation is present in approximately 3–4% of cases of mCRC and is associated with a poorer prognosis in terms of progression‐free survival (PFS) and OS compared to other KRAS mutations, as well as KRAS wild‐type tumors. In the AIO Colorectal Cancer Study Group pooled analysis of patients with mCRC receiving first‐line therapy, KRAS G12C was associated with the poorest OS, with an average of 16.8 months [11]. The results observed in the US, Japanese, and Italian cohorts were comparable, with a shorter PFS and OS in patients with KRAS G12C mutations relative to those with non‐G12C KRAS mutations [20, 28, 29]. As KRAS G12C mutant mCRC is now recognized as a specific, potentially treatable subset of mCRC, there is a need to ascertain the prevalence in larger, real‐world databases [30, 31]. Our study represents one of the most extensive real‐world KRAS G12C frequency datasets to date.

In Turkey, studies on RAS mutations in CRC have typically involved relatively small patient cohorts, with mutation frequency ranges reported between 11% and 49.1% [32, 33, 34, 35, 36]. One study involving 172 patients with mCRC identified a KRAS mutation frequency of 44%, with the most prevalent mutations being G12D (8.6%), G12V (8.2%), and G12C (2.3%) [32]. This study concentrated on seven specific mutations in codons 12 and 13 of the KRAS gene. Another study analyzed 220 colorectal tumor tissues from patients with both early‐stage and metastatic disease, finding an overall KRAS mutation frequency of 33.2% [33]. The most frequently identified mutations were G12D (14%), G12V (10%), and G12C (4%). The genomic DNA samples were assessed for mutations in codons 12 (G12A/C/D/F/R/S/V), 13 (G13A/C/D/R/S/V), and 61 (Q61E/H/K/L/P/R) of the KRAS gene. Notably, NRAS mutations were not examined in either study.

The wide‐scale molecular profiling conducted in this study holds significant clinical implications. First, our data provide a robust reference for KRAS and NRAS mutation frequencies in a large, real‐world Turkish mCRC population—an area where previously only limited and heterogeneous data existed. This not only supports more precise treatment planning on a national level but also contributes to global efforts in understanding regional variability in actionable mutations. Importantly, the identification of KRAS G12C mutations in 7.0% of KRAS‐mutant cases (3.4% of the entire cohort) highlights a clinically actionable subgroup. Recent clinical trials have demonstrated promising outcomes with KRAS G12C inhibitors (e.g., sotorasib and adagrasib) alone or in combination with anti‐EGFR therapy, suggesting a paradigm shift in the treatment of these patients [30, 31]. Recognizing and quantifying this subset in a large population is therefore essential to expand access to targeted therapies and clinical trials. Additionally, the uniform testing approach applied in our study also serves as a model for national diagnostic standardization in molecular oncology.

Given the high level of genetic diversity in Turkey, which includes European, Middle Eastern, and Asian populations, a comprehensive genetic analysis has been lacking. This underscores the need for detailed RAS molecular profiling to guide effective treatment strategies in Turkish mCRC patients, as mutation frequencies may vary significantly due to the country's ethnic heterogeneity. Figure 1A,B provide a geographic visualization of the distribution of KRAS/NRAS mutations and wild‐type cases across various provinces in Turkey. The highest numbers of both mutant and wild‐type cases are observed in major metropolitan regions such as Istanbul, Ankara, and Izmir, likely reflecting the population density and the presence of advanced oncology centers in these areas. However, the variation in mutation frequencies across different regions may also be influenced by the underlying ethnic diversity of the Turkish population. Turkey is home to multiple ethnic groups, including Turks, Kurds, Arabs, Zazas, Circassians, Bosniaks, Georgians, Greeks, Armenians, and Jews. The genetic background of these groups—shaped by diverse ancestral origins across Europe, the Middle East, and the Caucasus—may contribute to regional differences in mutation patterns observed in mCRC patients. Although the current study did not directly assess the ethnic background of patients, the observed geographical variation may indirectly reflect these differences. For instance, regions in eastern and southeastern Turkey—where ethnic minorities such as Kurds and Arabs are more densely represented—show distinct mutation‐to‐wild‐type ratios compared to the western regions. This suggests a possible genetic component influenced by ethnic ancestry, consistent with prior studies that have reported interethnic variability in RAS mutation frequencies. These findings underscore the importance of region‐specific and, ideally, ethnicity‐informed molecular profiling in mCRC patients. A more comprehensive understanding of the mutational landscape at the ethnic subgroup level could enhance precision oncology efforts by allowing for tailored therapeutic strategies. Future studies incorporating ethnic and genomic background data are warranted to clarify these correlations and support equitable, data‐driven treatment decisions across the country's diverse population.

This study involved the collection and analysis of tumor tissue samples from adult patients (≥ 18 years of age at diagnosis) diagnosed with mCRC. The samples were anonymized and collected from different centers in Turkey. However, apart from the diagnosis of mCRC, no demographic, clinical, tumor staging, or treatment‐related data (e.g., biopsy vs. surgical specimen distribution, chemotherapy administration before metastatic stage data) were available. Therefore, it was not possible to analyze the potential effects of prior treatment on mutation prevalence. Due to the retrospective and anonymized nature of the dataset, no demographic, clinical, survival, or treatment background data were available. This lack of data prevented us from analyzing the association between RAS mutation status and OS or PFS and from assessing the potential impact of prior therapies on mutation prevalence. The main limitation of this study is its reliance on PCR‐based methods rather than next‐generation sequencing. In addition, other clinically relevant biomarkers such as RAS mutations outside of hot codons, microsatellite instability, POLE mutations, and HER2 alterations were not analyzed in this study. Furthermore, KRAS and NRAS mutations are generally considered to be mutually exclusive, although rare co‐mutations have been reported. Because NRAS analysis was performed only in KRAS wild‐type cases, some co‐mutations may have been missed. Despite these limitations, our findings provide valuable insights into the molecular characteristics of mCRC in Turkey and contribute to the growing body of knowledge on precision oncology. Our data represent one of the most comprehensive single‐center real‐world RAS mutation datasets and contribute significantly to the understanding of the genetic landscape of mCRC.

## 5 Conclusion

The analysis of 10 754 samples from mCRC patients revealed a mutation frequency of 51.1% in the KRAS and NRAS genes, with 46.6% having KRAS mutations, 4.5% having NRAS mutations, and 48.5% being wild‐type for both. These results provide valuable real‐world data highlighting the importance of molecular testing in guiding effective treatment strategies and contribute significantly to the understanding of the genetic landscape of mCRC in Turkey.

## Conflict of interest

IA is employed by Heliks R&D and Biotechnology Inc. and owns stock in Heliks R&D and Biotechnology Inc. YHO, HB, and HK are employed by Genetiks Genetic Diagnostic Center Inc. and own stock in Genetiks Genetic Diagnostic Center Inc. The remaining authors declare no competing interests.

## Author contributions

Conception/Design: GK, IA, YHO, HB, HK, OD, SY. Data Analysis and Interpretation: GK, IA, YHO, HB, HK. Manuscript Writing: GK, OD, SY. Final Approval of Manuscript: GK, IA, YHO, HB, HK, OD, SY.

## Data Availability

The datasets analyzed during this study are available from the corresponding author upon reasonable request.
